# Development of Chinese Version of Polycystic Ovary Syndrome Health-Related Quality of Life Questionnaire (Chi-PCOSQ)

**DOI:** 10.1371/journal.pone.0137772

**Published:** 2015-10-09

**Authors:** Huang-tz Ou, Meng-Hsing Wu, Chung-Ying Lin, Pei-Chi Chen

**Affiliations:** 1 Institute of Clinical Pharmacy and Pharmaceutical Sciences, National Cheng Kung University College of Medicine, Tainan, Taiwan; 2 Department of Obstetrics and Gynecology, National Cheng Kung University College of Medicine and Hospital, Tainan, Taiwan; 3 Department of Public Health, National Cheng Kung University College of Medicine, Tainan, Taiwan; University of Sydney, AUSTRALIA

## Abstract

**Objectives:**

To develop the Chinese version of the Polycystic Ovary Syndrome Health-related Quality of Life Questionnaire (Chi-PCOSQ).

**Research Design and Method:**

This cross-sectional study was conducted in a medical center in Taiwan. Eighty women who met the criteria were enrolled: female, age range of 18–45 years, competent in the Chinese language, had been diagnosed with polycystic ovary syndrome (PCOS), and were regularly followed at outpatient clinics (defined as at least two outpatient visits before enrollment). The PCOSQ was translated and culturally adapted according to standard procedures. A semi-structured interview was applied to assess face validity. Exploratory factor analysis (EFA) was applied to determine scale constructs. Measurements of internal consistency via Cronbach’s α, test-retest reliability via intraclass correlation coefficient (ICC), construct validity, and discriminative validity were performed.

**Results:**

Five additional items, representing the issues of acne, hair loss, and fear of getting diabetes, were incorporated into the original scale. A six-factor structure emerged as a result of the EFA, explaining 71.9% of the variance observed. The reliability analyses demonstrated satisfactory results for Cronbach’s α ranging from 0.78–0.96, and for ICC ranging from 0.73–0.86. Construct validity was confirmed by significant correlation between the domains of the Chi-PCOSQ and generic health-related quality of life (HRQoL) measures (WHOQOL-BREF, EQ-5D) and clinical parameters (body mass index, waist-hip ratio, blood pressure). The known-group analysis indicated that the Chi-PCOSQ is a discriminative tool that differentiates patients according to their HRQoL.

**Conclusion:**

The Chi-PCOSQ seems internally consistent, culturally acceptable, and our preliminary evidence suggests that it may be reliable and valid. The Chi-PCOSQ is a promising assessment tool to address the HRQoL of women affected by PCOS in Chinese-speaking countries and to further identify ethnic/cultural differences in the HRQoL of women with PCOS.

## Introduction

Polycystic ovary syndrome (PCOS) is a common endocrine disorder, affecting approximately 6–10% of reproductive-age women [1]. The symptoms typically associated with PCOS (e.g., amenorrhea, oligomenorrhea, hirsutism, obesity, infertility, anovulation, and acne) can lead to a significant reduction in health-related quality of life (HRQoL), cause mood disturbances [2], and impact the emotional well-being as well as sexual satisfaction of women [3–6]. Typical clinical presentations of PCOS such as acne, hirsutism, and obesity, along with menstrual irregularity and infertility, have been found to significantly contribute to the psychological morbidity of patients [4, 7–11]. Despite this, little research, especially East Asian studies, has been carried out to examine the impact of the symptoms and associated treatments for PCOS on the HRQoL of patients. One of the main reasons is that no validated health outcome measure exists for measuring the health status of women with PCOS. Although some may argue that generic questionnaires such as the Short Form 36 (SF-36) [12] and World Health Organization Quality of Life Questionnaire (WHOQOL) [13] can be used, these two questionnaires are not specifically designed for patients with PCOS, and may only provide healthcare providers limited HRQoL information.

HRQoL has been recognized as a multidimensional concept, including physical, emotional, and social aspects associated with a specific disease or its treatment [14]. Assessing HRQoL provides important information on the benefits of medical therapies or interventions from the patients’ perspective [15]. Two main approaches, namely generic and disease-specific instruments, can be applied to measure HRQoL in PCOS patients. Generic instruments, such as the SF-36 [12] and WHOQOL [13], have been applied to women with PCOS [7, 10, 11, 16–21]. However, generic instruments that are designed to measure HRQoL over a broad spectrum of diseases may not be sensitive enough to detect HRQoL specific to a particular illness of interest [22]. For example, the problems of weight and infertility, which are significantly affected by PCOS, would not be identified when using a generic measurement (e.g., WHOQOL) because generic instruments typically have no items pertaining to these PCOS-specific issues.

Although generic HRQoL instruments are most commonly used in patients with PCOS, there is one PCOS-specific instrument currently available, namely the Polycystic Ovary Syndrome Health-related Quality of Life Questionnaire (PCOSQ) [23]. It aims to measure the symptoms and psychological impacts of PCOS. The PCOSQ, originally developed in the United States (US) [23], has been validated in other English-speaking countries such as the United Kingdom (UK) [21] and Canada [6]. The modified PCOSQ (mPCOSQ), developed by Barnard et al. [5], adds 6 items that assess acne problems into the original scale, which consists of 24 items, for a total of 30 items that represent 6 subscales. Its psychometric properties have been evaluated in Iranian women [24]. Although the PCOSQ has been recently translated into Persian and validated for Iranian women [24] and adapted for Brazilian women [25], it is mostly used in English-speaking countries: US [4], UK [17, 26] and Australia [27]. Therefore, there is a lack of PCOSQ versions in other languages, especially for countries that are ethnically and culturally different from the West.

The impact of PCOS on HRQoL may depend on ethnicity and culture [28]. Hence, it is important to determine the appropriateness of PCOSQ use in Asia. There is a need for HRQoL measures specifically for use in non-English-speaking countries because cultural groups vary in clinical representations of PCOS, which may be attributable to HRQoL varying with ethnicity [28]. Previous studies that used cross-cultural comparisons demonstrated differences in the HRQoL of PCOS women between women in the UK and those living there of South Asian origin [26], between Austrians and Muslim immigrants [27], and between Austrians and Brazilians [25]. Moreover, it is recognized that women affected by PCOS have increased long-term risks of development of type 2 diabetes, cardiovascular diseases, and cancer. There is a high prevalence of metabolic syndrome and associated diseases among women with PCOS in Asia [29, 30]. Individuals’ fears of getting diabetes may affect their psychological well-being, which should be considered in the assessment of HRQoL, particularly in Asian women with PCOS. However, this concept is not included in the existing PCOS-specific HRQoL assessment (i.e., PCOSQ). This is in part due to a lack of previous research on the HRQoL of East Asian women affected by PCOS. Therefore, the present study aims to develop a Chinese version of the PCOSQ (Chi-PCOSQ).

## Materials and Methods

### Participants

Participants from National Cheng Kung University Hospital had all attended the Department of Obstetrics & Gynecology and met the inclusion criteria: (1) female, (2) aged 18–45 years, (3) competent in the Chinese language, (4) had a diagnosis of PCOS, and (4) were regularly (at least two outpatient visits before enrollment) followed at outpatient clinics. Exclusion criteria were: (1) diagnosed with a similar clinical presentation, including congenital adrenal hyperplasia, androgen secreting tumors, Cushing syndrome, thyroid dysfunction, and hyperprolactinaemia, (2) previously diagnosed diabetes or Fasting Plasma Glucose Test > 126 mg/dL at the time of inclusion, (3) took any medication having an effect on insulin levels or hormonal medications, including contraceptive pills, at least two months before participating this study, (4) known to have suffered a major traumatic event at least 6 months prior to data collection, such as divorce, separation, or death of someone close.

All participants provided written informed consent regarding their willingness to participate in the research. A well-trained interviewer assessed each participant, and confirmed that all participators completed the Chi-PCOSQ, WHOQOL-BREF, EQ-5D, and demographic questions regarding age, gender, residence, highest education level, disease duration, and disease subtype.

### Measurements

**PCOSQ** [23] is a disease-specific HRQoL questionnaire that contains 26 questions with a seven-point rating scale (1: maximum impairment and 7: no impairment of HRQoL) in the following five domains: emotion (7 items), hair growth (5 items), body weight (5 items), infertility (5 items), and menstruation (4 items). The psychometric properties of the PCOSQ show good test-retest reliability (all intraclass coefficients > 0.8), acceptable internal reliability (all Conbroach’s α values > 0.7), and satisfactory concurrent validity with the SF-36 [21]. The responsiveness of the PCOSQ was also established in a randomized controlled trial of troglitazone [6]. The PCOSQ was responsive to treatment effects. HRQoL was found to be improved for the domains that corresponded to the treated symptoms (e.g., infertility, emotion, and menstruation), and no improved HRQoL was found for the domains without treatment effects (e.g., weight and hirsutism).

**WHOQOL-BREF** [31] is a short-form version of the WHOQOL-100 and has 26 items (the WHOQOL-BREF Taiwan version additionally includes two domestic items) distributed into two generic items and four domains: physical health (7 items), psychological health (6 items), social relations (4 items), and environment (11 items). The internal consistency (Cronbach’s α = 0.70–0.91), the test-retest reliability (r = 0.76–0.80), and the construct validity (comparative fit index [CFI] = 0.89) have been established for the Taiwan version scores [32].

**5-dimension EuroQoL questionnaire (EQ-5D)** is a multi-attribute utility instrument with five dimensions (mobility, self-care, usual activities, pain/discomfort, and anxiety/depression), each of which has three levels of severity (health states). The Taiwan version was validated in a previous study [33]. Using the scoring function from Taiwan, these health state parameters were transformed into a utility value ranging from 0 to 1, where 0 represents death and 1 indicates full health.

### Translating PCOSQ into Chinese and statistical analyses

Permission for the study from the Institutional Review Board of the National Cheng Kung University Hospital was obtained before study commencement (A-ER-103-287). The User Agreement and Translation Agreement of the English version of the PCOSQ was obtained from the original authors [23]. The translation process was guided by an expert panel, which included two coordinators (one researcher and one physician), one psychometric expert, four translators, and two interviewers.

#### Forward and Back Translations

Two Chinese translators (one was a physician and the other had no medical background) proficiently fluent in English independently translated the complete English version of the PCOSQ, including item content, response options, and instructions, into Chinese. The two forward translations were merged into one version after discussions of the expert panel.

All the experts considered the Chinese version relatively consistent with the original PCOSQ. However, certain items required discussion. First, many sentences in the original items used the term “problem”, which can be directly translated into Chinese as “問題” (pinyin: *wen ti*). However, the experts were concerned that this translated term may be too strong and even provoke uncomfortable feelings. Therefore, a more gentle Chinese term “困擾” (pinyin: *kun rao*) was used instead. Second, for Q18 “self-conscious as a result of having PCOS?”, the expert panel could not find an appropriate Chinese term that corresponds to “self-conscious.” As a result, the term “敏感” (pinyin: *min gan*, meaning sensitive) was used instead, and an additional explanation sentence was provided: “例如:覺得容易變成注視或討論的對象” (pinyin: *li ru*: *jue de rong yi bian cheng zhu shi huo tao lun de dui xiang*, meaning: for example, feel that people are looking at you or talking about you). Third, for Q11 “had low self-esteem as a result of having your PCOS?”, the direct translation of “low self-esteem” into Chinese as “低自尊” (pinyin: *di zi zun*) may not be easily understood, so the experts decided to use a similar meaning term in Chinese “自卑” (pinyin: *zih bei*, meaning self-abased).

In backward translation, two translators (one was a nurse and the other had no medical background) with a high level of fluency in English independently translated the single forward translation back into English while totally blinded to the original English version of the PCOSQ. With the help of a translation coordinator, the agreements and differences between the backward English translations and the original questionnaire were identified. The experts agreed that the translated-back items in both versions were fully consistent with the meaning of the original items. Also, the experts acknowledged the relationship between metabolic syndrome and PCOS in Asian women [29, 30] and thus decided to add one item that measured the worries of the patients regarding developing diabetes. Therefore, there were 27 items for the pilot test. All translations are available upon request.

#### Pilot test and identification of culturally specific issues in PCOS patients

We interviewed 22 PCOS patients with different educational levels (high school, college, and graduate school level) individually using a semi-structured questionnaire. The interview focused on items that were difficult, confusing, or offensive and patients were asked about any concerns about their HRQoL that may be under-represented in the original PCOSQ. The pilot respondents also reported the importance of the original 26 items on the PCOSQ and the additional item regarding worries toward diabetes (Q14_2).

All respondents indicated that all 27 items were relevant to their concerns regarding the effects of PCOS on their HRQoL. They had no difficulties understanding the meaning of items and interpreting the items using their own words. However, acne and hair loss, which were not included in the original PCOSQ, were the most common reported issues by the pilot participants. Therefore, the expert panel decided to add the issues of “acne” and “hair loss” as culturally specific concepts, which resulted in four additional question items, phrased as “growth of visible acne (or excess hair loss)” and “feel that visible acne (or excess hair loss) is a problem”. Finally, 31 items were used for field testing.

#### Field testing, psychometric validation, and statistical analysis of Chi-PCOSQ

Descriptive analyses were used for demographics and the PCOSQ item scores. Exploratory factor analysis (EFA) using principle component analysis with varimax rotation was conducted to examine the factor structure of the Chi-PCOSQ. The original PCOSQ contains five domains [23], and the Chi-PCOSQ contains one additional domain that includes the new items of acne and hair loss issues. Therefore, a six-factor solution was used for the Chi-PCOSQ. Eigenvalues and percentages of the explained variance were calculated for the six domains. We expected the items to have a factor loading of > 0.4. In addition, items Q11, Q18, Q6, Q2, Q17, Q14_1, Q14_2, Q4, and Q23 should be loaded on the emotion domain; items Q24, Q3, Q12, Q10, and Q22 should be loaded on the weight domain; items Q16, Q26, Q15, Q9, and Q1 should be loaded on the body hair domain; items Q5, Q25, and Q13 should be loaded on the infertility domain; items Q19, Q21, Q20, Q8, and Q7 should be loaded on the menstrual domain; and items Q29, Q30, Q27, and Q28 (i.e., new items) should be loaded on the new domain. The recommended sample size for EFA is either *n* ≥ 100 or a > 2:1 ratio of subjects to items to reduce statistical error [21, 34]. Because the Chi-PCOSQ contains 31 items, the sample size should be more than 62 subjects.

Internal consistency using Cronbach’s α was used for each domain, and the item-total correlations of all items were calculated. An α value of > 0.70 suggests acceptable internal reliability, and an item with an item-total correlation of > 0.4 indicates that the item is adequate. Moreover, Pearson correlation was used for test-retest reliability (for an interval of two weeks to one month), with a minimum reliability threshold of 0.70 used as a measure of adequate test-retest results.

After the factor structure of the PCOSQ and its reliability had been examined, validity evaluations, including construct validity and known-group validity, were conducted to additionally explore its psychometric properties. Construct validity was tested using the following standards: two generic HRQoL questionnaires (i.e., EQ-5D and WHOQOL-BREF) and four physiological indicators (i.e., BMI, waist-hip ratio (WHR), systolic blood pressure, diastolic blood pressure). As for known-group validity, we compared the PCOSQ domain scores among the following groups: participants with sexual experiences vs. those without sexual experiences; participants with acne problems vs. those without acne problems; participants with hair loss problems vs. those without hair loss problems; participants with at least one family member having diabetes vs. those without any family members having diabetes.

## Results

The mean (standard deviation, SD) age of the pilot participants (n = 22) was 29.50 (SD: 5.76) years, and that of all participants was 28.00 (SD: 5.57) years. In addition, one pilot participant had a senior high school degree, 18 had college degrees, and 3 had graduate degrees. One eligible person refused to participate, leaving a total of 80 enrolled participants for data analyses. 11 participants had senior high school degrees, 56 had college degrees, and 13 had graduate degrees. Almost all participants did not smoke currently (n = 78), more than half of them did not drink currently (n = 55), and most of them did not regularly exercise (77 exercised twice or fewer times per week). About 38% of the participants had at least one family member with diabetes, and 70% of the participants had sexual experiences. The mean BMI (26.26) and mean WHR (0.80) were slightly higher than the standard values. Acne and hair loss problems were reported by more than half of the participants. In addition, the mean of systolic blood pressure was 119.52 (SD: 17.03) mmHg and that of diastolic blood pressure was 77.16 (SD: 14.05) (Table 1).

**Table 1:** Participants’ characteristics.

	Mean or (n)	Standard deviation or (%)
Age (year)	28.00	5.57
Educational level		
Senior high	(11)	(13.8%)
College	(56)	(70.0%)
Graduate	(13)	(16.3%)
Current smoking status (No)	(78)	(97.5%)
Current drinking status (No)	(55)	(68.8%)
Exercise habit		
< 1 time/week	(44)	(55.0%)
1–2 times/week	(33)	(41.3%)
> 2 times/week	(3)	(3.7%)
Family history of diabetes (No)	(30)	(37.5%)
Sexual experience (No)	(24)	(30.0%)
Height (cm)	158.93	5.64
Weight (kg)	66.49	17.47
Body mass index (m^2^/kg)	26.26	6.56
Waist (cm)^a^	81.97	13.40
Hip (cm)^a^	102.47	11.13
Waist-hip ratio ^a^	0.80	0.06
Acne (No)^b^	(31)	(41.3%)
Hair loss (No)	(32)	(43.2%)
Systolic blood pressure (mmHg)^c^	119.52	17.03
Diastolic blood pressure (mmHg)^c^	77.16	14.05

^a^ n = 77

^b^ n = 75

^c^ n = 79

The pilot participants rated the importance of each item. About one fourth and one third of them suggested that hair loss and acne, respectively, should be considered when measuring their HRQoL (Table 2). Scores of each Chi-PCOSQ item from the pilot and all participants were similar.

**Table 2:** Chi-PCOSQ scores and item importance.

	Mean (standard deviation)		
Item # and description	Importance*: pilot (n = 22)	Score**: pilot (n = 22)	Score**: all (n = 80)
Q2 Depressed as a result of having PCOS	2.40 (1.20)	3.50 (2.00)	3.46 (1.99)
Q3 Concerned about being overweight	4.20 (1.20)	0.90 (1.40)	1.71 (1.82)
Q4 Tired easily	3.50 (1.20)	1.80 (1.60)	2.53 (1.53)
Q5 Concerned about infertility problems	2.90 (1.80)	2.70 (2.60)	2.09 (1.97)
Q6 Moody as a result of having PCOS	2.80 (1.10)	3.20 (2.00)	3.49 (1.84)
Q10 Trouble dealing with weight	4.23 (1.24)	1.23 (1.69)	2.06 (2.00)
Q11 Low self-esteem as a result of PCOS	1.92 (1.19)	4.31 (1.84)	4.28 (1.96)
Q12 Frustration in trying to lose weight	3.92 (1.26)	1.54 (1.90)	2.59 (2.15)
Q13 Afraid of not being able to have children	3.00 (1.87)	2.85 (2.58)	2.29 (2.08)
Q14_1 Afraid of getting cancer	2.54 (1.51)	3.46 (2.11)	2.79 (2.18)
Q14_2 Afraid of getting diabetes	3.62 (1.39)	2.38 (1.81)	2.38 (2.02)
Q17 Worried about having PCOS	2.92 (1.61)	2.54 (2.26)	2.71 (1.82)
Q18 Self-conscious as a result of having PCOS	2.15 (1.14)	4.00 (2.00)	4.24 (1.85)
Q22 Feel unsexy because overweight	3.31 (1.49)	2.15 (2.15)	3.26 (2.17)
Q23 Lack of control over the situation with PCOS	3.08 (1.38)	2.69 (1.97)	2.91 (2.06)
Q24 Difficulties staying at your ideal weight	3.62 (1.56)	1.31 (1.75)	2.05 (1.98)
Q25 Sad because of infertility problems	2.62 (1.76)	3.69 (2.25)	2.77 (2.08)
Q1 Growth of visible hair on chin	1.80 (1.40)	4.90 (1.90)	5.06 (1.46)
Q9 Growth of visible hair on upper lip	1.80 (1.40)	4.60 (1.90)	4.72 (1.71)
Q15 Growth of visible hair on your face	1.85 (1.21)	4.92 (1.66)	4.46 (1.84)
Q16 Embarrassment about excessive body hair	2.31 (1.38)	4.46 (1.85)	4.22 (2.04)
Q26 Growth of visible body hair	2.54 (1.51)	3.54 (2.47)	3.92 (2.12)
Q7 Headaches	3.00 (1.60)	3.60 (2.30)	3.97 (1.89)
Q8 Irregular menstrual periods	3.80 (1.40)	1.40 (2.10)	1.50 (2.03)
Q19 Abdominal bloating	2.85 (1.52)	3.31 (1.93)	3.11 (1.96)
Q20 Last menstruation period	3.46 (1.71)	1.92 (2.43)	2.20 (2.30)
Q21 Menstrual cramps	3.00 (1.58)	3.15 (2.23)	3.45 (2.17)
Q27 ^a^ Excess hair loss	5	—	3.06 (2.10)
Q28 ^a^ Feel excess hair loss is a problem	5	—	3.49 (2.12)
Q29 ^a^ Growth of visible acne	7	—	3.50 (2.13)
Q30 ^a^ Feel acne is a problem	7	—	3.71 (2.08)

* Importance rating on each item ranges from 1 to 5, representing Not Important to Very Important.

** Individual items are scored from 1 to 7, where 1 indicates maximum impairment and 7 indicates no impairment of HRQoL.

^a^ Number of pilot participants that reported that this is an important item.

According to the suggestions from pilot participants, four items were added to the Chi-PCOSQ. A total of 31 items were examined for psychometric properties. Because the four additional items were related to acne and hair loss, we named the underlying domain of the four items as the acne and hair loss domain. Moreover, using a cut-off value of 0.40 for factor loadings, EFA showed that all items loaded on the expected domains, except for items Q14_2 and Q4. Item Q14_2 cross-loaded on the weight domain (loading = 0.469), and item Q4 loaded on the acne and hair loss domain (loading = 0.503). Therefore, items Q14_2 and Q4 were included in the weight and acne and hair loss domains, respectively, in the following analyses.

The Chi-PCOSQ was found to have six factors. The eigenvalues of the six domains were all > 1, with six factors explaining 71.88%. The discrepancy was ‘Q4: tired easily’, which loaded on the acne and hair loss domain in the current research and on the emotion domain in its original scale. Internal consistency was high for the entire Chi-PCOSQ (α = 0.939) and those of the six domains were all acceptable (α > 0.70). The item-total correlations of items were all satisfactory (r > 0.40). All domains and items had adequate test-retest reliability (Table 3).

**Table 3:** Internal consistency, item-total correlation, and test-retest reliability. Note: Cronbach’s α for total score of PCOSQ = 0.939; values for constructs are in **bold**.

	Eigenvalue, explained %	Factor loading	Cronbach’s α	Item-total correlation	Test- retest
**Construct: Emotion**	**11.238, 36.251%**		**0.897**		**0.861**
Q11 Low self-esteem as a result of PCOS		0.793		0.744	0.701
Q18 Self-conscious as a result of having PCOS		0.785		0.756	0.794
Q6 Moody as a result of having PCOS		0.750		0.748	0.747
Q2 Depressed as a result of having PCOS		0.748		0.722	0.713
Q17 Worried about having PCOS		0.653		0.756	0.799
Q14_1 Afraid of getting cancer		0.576		0.528	0.788
Q23 Lack of control over the situation with PCOS		0.528		0.680	0.765
**Construct: Weight**	**3.452, 11.136%**		**0.914**		**0.860**
Q24 Difficulties staying at your ideal weight		0.874		0.851	0.763
Q3 Concerned about being over weight		0.873		0.817	0.757
Q12 Frustration in trying to lose weight		0.866		0.849	0.794
Q10 Trouble dealing with weight		0.860		0.850	0.816
Q22 Feel unsexy because overweight		0.713		0.710	0.752
Q14_2 Afraid of getting diabetes		0.469		0.504	0.698
**Construct: Body hair**	**2.507, 8.086%**		**0.910**		**0.779**
Q16 Embarrassment about excessive body hair		0.822		0.818	0.719
Q26 Growth of visible body hair		0.811		0.765	0.768
Q15 Growth of visible hair on your face		0.810		0.810	0.765
Q9 Growth of visible hair on upper lip		0.788		0.774	0.671
Q1 Growth of visible hair on chin		0.768		0.733	0.644
**Construct: Infertility**	**2.230, 7.193%**		**0.960**		**0.825**
Q5 Concerned about infertility problems		0.863		0.920	0.812
Q25 Sad because of infertility problems		0.852		0.920	0.689
Q13 Afraid of not being able to have children		0.846		0.908	0.830
**Construct: Acne & hair loss**	**1.677, 5.410%**		**0.853**		**0.834**
Q29 Growth of visible acne		0.788		0.691	0.855
Q30 Feel acne is a problem		0.765		0.753	0.777
Q27 Excess hair loss		0.721		0.673	0.734
Q28 Feel excess hair loss is a problem		0.714		0.726	0.678
Q4 Tired easily		0.503		0.491	0.525
**Construct: Menstrual**	**1.179, 3.802%**		**0.782**		**0.726**
Q19 Abdominal bloating		0.860		0.550	0.606
Q21 Menstrual cramps		0.744		0.484	0.711
Q20 Last menstruation period		0.651		0.678	0.445
Q8 Irregular menstrual periods		0.488		0.600	0.568
Q7 Headaches		0.486		0.494	0.627

Before examining the construct validity, the domain scores were calculated as the sums of the item scores divided by the number of items. Taking the emotion domain as an example, the sum of the 7 item scores was divided by 7. Therefore, the score for each domain was in the range of 1–7. Table 4 indicated that all domain scores of the Chi-PCOSQ were significantly correlated with EQ-5D score (r = 0.294 to 0.470). Body hair, acne and hair loss, and menstrual domains were significantly correlated with the physical domain of the WHOQOL-BREF (r = 0.335 to 0.532). Emotion, weight, acne and hair loss, and menstrual domains were significantly correlated with the psychological domain of the WHOQOL-BREF (r = 0.293 to 0.493). Emotion, weight, body hair, acne and hair loss, and menstrual domains were significantly correlated with the social domain of the WHOQOL-BREF (r = 0.247 to 0.519). Emotion, body hair, infertility, acne and hair loss, and menstrual domains were significantly correlated with the environment domain of the WHOQOL-BREF (r = 0.231 to 0.521). In addition, only the weight domain score of the PCOSQ was significantly correlated with participants’ BMI (r = −0.594), WHR (r = −0.352), and blood pressure (r = −0.254 and −0.255).

**Table 4:** Construct validity for Chi-PCOSQ. Note: BMI = body mass index; WHR = waist-hip ratio; SBP = systolic blood pressure; DBP = diastolic blood pressure.

	*r* (*p*)					
	Emotion	Weight	Body hair	Infertility	Acne and hair loss	Menstrual
EQ-5D (n = 80)	0.408 (< 0.001)*	0.301 (0.007)*	0.297 (0.008)*	0.294 (0.008)*	0.349 (0.002)*	0.470 (<0.001)*
WHOQOL (n = 80)						
Physical	0.218 (0.052)	0.205 (0.07)	0.335 (0.002)*	−0.003 (0.98)	0.532 (<0.001)*	0.405 (<0.001)*
Psychological	0.293 (0.008)*	0.396 (<0.001)*	0.217 (0.053)	0.081 (0.47)	0.493 (<0.001)*	0.295 (0.008)*
Social	0.386 (<0.001)*	0.250 (0.025)*	0.331 (0.003)*	0.108 (0.34)	0.519 (<0.001)*	0.247 (0.03)*
Environment	0.347 (0.002)*	0.187 (0.10)	0.232 (0.04)*	0.231 (0.04)*	0.521 (<0.001)*	0.429 (<0.001)*
BMI (n = 80)	−0.176 (0.12)	−0.594 (<0.001)*	−0.069 (0.54)	−0.063 (0.58)	−0.044 (0.70)	−0.042 (0.71)
WHR (n = 77)	−0.093 (0.42)	−0.352 (0.002)*	0.039 (0.74)	0.099 (0.39)	−0.036 (0.76)	0.079 (0.50)
SBP (n = 79)	−0.254 (0.02)*	−0.494 (<0.001)*	−0.101 (0.38)	−0.036 (0.75)	−0.178 (0.12)	−0.066 (0.56)
DBP (n = 79)	−0.255 (0.02)*	−0.410 (<0.001)*	0.010 (0.93)	−0.112 (0.32)	−0.175 (0.12)	−0.032 (0.78)

* *p* <0 .05

Significant differences were found in the infertility domain between participants with sexual experiences (1.86 ± 1.82) and those without (3.61 ± 1.77; p < .001); in the acne and hair loss domain between participants with acne problems (2.64 ± 1.51) and those without (4.06 ± 1.21; p < 0.001) and between those with hair loss problems (2.70 ± 1.51) and those without (4.07 ± 1.33; p < 0.01); in the menstrual domain between participants whose family member had a history of diabetes (2.57 ± 1.45) and those whose family members did not have a history of diabetes (3.31 ± 1.53; *p* = 0.035) (Table 5).

**Table 5:** Known-group validity for Chi-PCOSQ.

	Domain score: Mean (standard deviation)					
	Emotion	Weight	Body hair	Infertility	Acne and hair loss	Menstrual
**Sexual experience**						
No (n = 24)	3.64 (1.48)	2.39 (1.64)	4.27 (1.40)	3.61 (1.77)	3.50 (1.55)	3.59 (1.45)
Yes (n = 56)	3.32 (1.57)	2.32 (1.73)	4.57 (1.66)	1.86 (1.82)	3.15 (1.61)	2.53 (1.44)
t (p)	0.836 (0.41)	0.162 (0.87)	0.755 (0.45)	3.981 (<0.001)*	0.891 (0.38)	3.018 (0.003)*
**Acne**						
No (n = 31)	3.45 (1.64)	2.47 (1.38)	4.65 (1.55)	2.03 (1.89)	4.06 (1.21)	2.97 (1.40)
Yes (n = 44)	3.33 (1.50)	2.18 (1.81)	4.44 (1.59)	2.67 (2.02)	2.64 (1.51)	2.64 (1.46)
t (p)	0.329 (0.74)	0.755 (0.45)	0.565 (0.57)	1.391 (0.17)	4.355 (<0.001)*	0.990 (0.33)
**Hair loss**						
No (n = 32)	3.80 (1.35)	2.67 (1.51)	4.89 (1.10)	2.81 (1.88)	4.07 (1.33)	3.09 (1.57)
Yes (n = 42)	3.18 (1.66)	2.15 (1.77)	4.30 (1.84)	2.10 (2.01)	2.70 (1.51)	2.56 (1.35)
t (p)	1.741 (0.09)	1.348 (0.18)	1.708 (0.09)	1.564 (0.12)	4.074 (<0.001)*	1.545 (0.13)
**Family history of diabetes**						
No (n = 30)	3.45 (1.57)	2.78 (2.07)	4.37 (1.70)	2.09 (2.05)	3.42 (1.47)	3.31 (1.53)
Yes (n = 50)	3.39 (1.54)	2.08 (1.37)	4.54 (1.52)	2.56 (1.92)	3.16 (1.67)	2.57 (1.45)
t (p)	0.165 (0.87)	1.663 (0.10)	0.464 (0.64)	1.037 (0.30)	0.705 (0.48)	2.145 (0.04)*

* *p* < 0.05

## Discussion

The symptoms of acne and hirsutism and the issues of irregular menses and infertility caused by PCOS interfere with daily activities and bring considerable anxiety and depression to patients. The PCOSQ is commonly applied in the West [4, 6, 17, 21, 23, 26] and the care support and people’s attitudes toward life may differ from those in East Asia. The assessment of HRQoL for PCOS patients is essential and thus we translated the PCOSQ and validated the translation for Chinese-speaking countries. This research makes an important contribution to the validation of the PCOSQ because of the incorporation of the acne, hair loss, and frightened of getting diabetes subscales into the amended scale, which is culturally appropriate in Chinese-speaking countries. Hence, the Chi-PCOSQ (S1 Appendix) provides an opportunity to compare attitudes towards PCOS issues between Western and Chinese cultures. The Chi-PCOSQ can improve our understanding of the impact that the symptoms and treatments for PCOS have upon patients’ HRQoL in East Asia.

### Comparison of HRQoL of women with PCOS between Chinese-speaking and other countries

**Fig 1:**
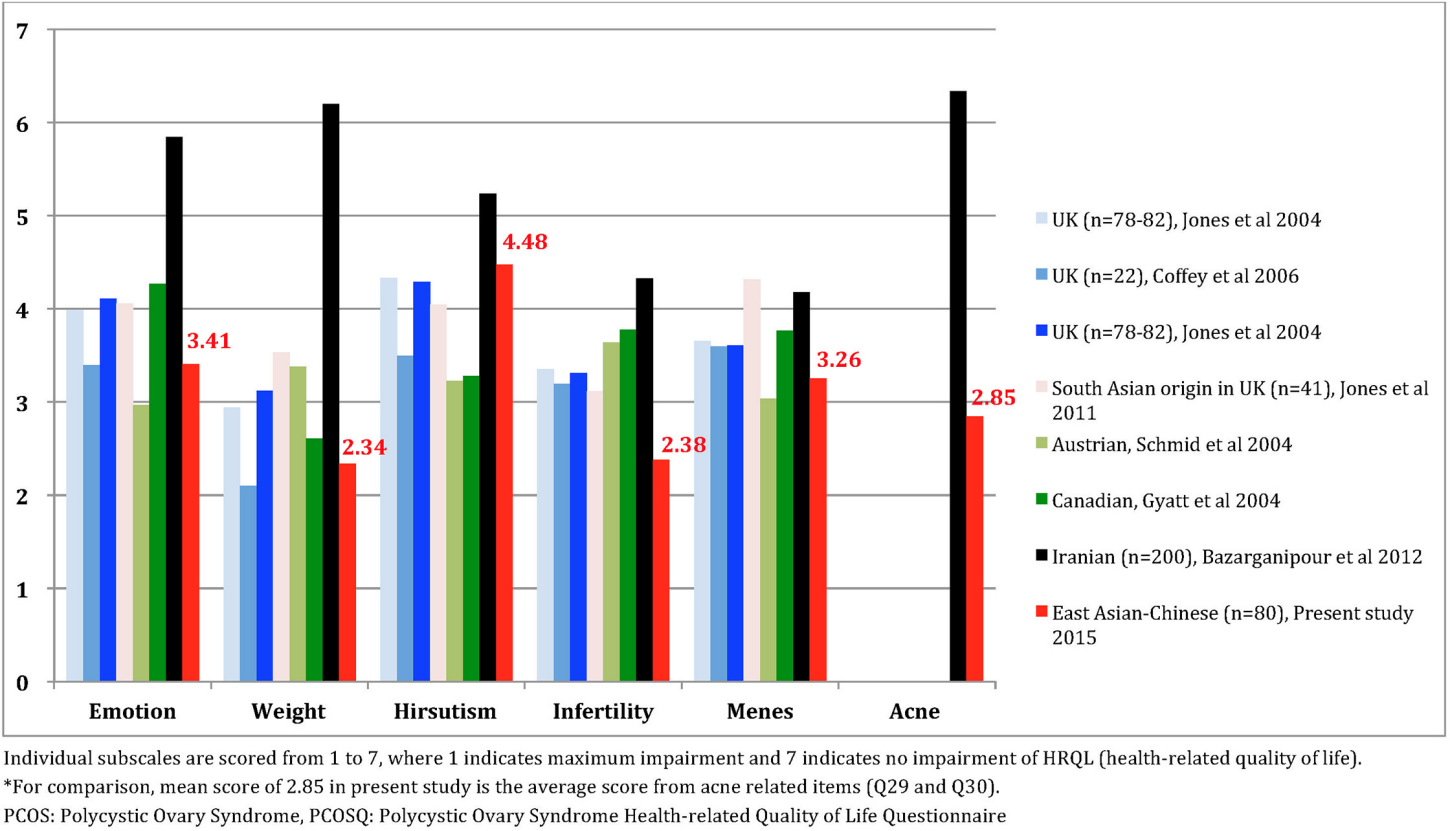
Comparisons across ethnicities in HRQoL of PCOS women measured using PCOSQ.

As compared to other ethnic origins, the clinical manifestations of PCOS on HRQoL in Iranian women in general are lesser extent (Fig 1). East Asian patients, as presented by our Chinese participants, struggled more with infertility and acne. However, the impact of hirsutism on the HRQoL of Chinese-speaking patients was trivial. This finding is consistent with previous studies on South Chinese, who were less hirsute and had lower Ferriman-Gallwey (F-G) scores [35]; fewer than 10% of Chinese patients had a modified F-G score of more than 5 [36]. Although previous studies had examined the impact of acne problems on PCOS patients’ HRQoL in the US [5] and Brazil [24], our results indicate that acne problems for Chinese patients may be more severe. Hence, the present study highlights the importance of managing emotional disturbances due to clinical manifestations of PCOSQ, especially infertility and acne problems, on the HRQoL of East Asians.

### Psychometric properties

Our rigorous translation procedure and psychometric evaluation ensured that the Chi-PCOSQ is scientific and convincing. All aspects of assessment indicate that the Chi-PCOSQ has good measurement properties, and the results of psychometric properties agree with previous validation studies of the PCOSQ [6, 21]. Although our proposed 6-factor solution structure for the Chi-PCOSQ is slightly different from the 5-factor solution structure in most previous studies [6, 21], we are confident that our structure is suitable for East Asians based on the following reasons. First, the factor loadings of individual items on the five dimensions of emotional disturbance, hirsutism, weight concerns, infertility, and menstrual problems were identical to those in previous findings [6, 21, 23], expect for one item, “tired easily”, which was loaded on the acne and hair loss domain. As for the five added items, “afraid of getting diabetes” was loaded on the weight domain, while the other four were loaded on the acne and hair loss domain. Second, our EFA results suggest that the factor loadings of five additional items (those related to acne, hair loss, and afraid of getting diabetes) on the corresponding domains are satisfactory. Although “tired easily” was loaded on the acne and hair loss domain, rather than on the emotion domain, as in the original scale, this loading is acceptable. Third, the internal-consistency of the Chi-PCOSQ was excellent in all six domains.

All the coefficients of the Chi-PCOSQ domains from test-retest reliability analysis were greater than 0.7, implying that the scale had satisfactory reproducibility. However, as to individual items, the coefficients of test-retest reliability values for “Q20: late menstrual period” and “Q8: irregular menstrual periods” were relatively low. This may be because most of the respondents had begun taking metformin after the baseline interview and most reported an improved menstrual cycle when they were back for their second interview (when the re-test was administrated). As a result, test-retest analysis for menstrual-related questions showed some inconsistency between the two tests.

In construct validity analysis, the Chi-PCOSQ was correlated with generic HRQoL measures (WHOQOL-BREF, EQ-5D) and clinical parameters (e.g., weight and blood pressure). Also, the Chi-PSOCQ had the ability to differentiate between patients according to their HRQoL at a point in time. In the known-group validity analysis, the Chi-PCOSQ provided support to all hypotheses depending upon known performances of the construct that the scale is intended to measure. This is, the respondents who had sexual experiences were more worried about infertility problems (as shown by lower scores for the infertility domain). Those experiencing the problem of acne or hair loss reported significantly lower scores for the acne and hair loss domain of the Chi-PCOSQ. Family history is important in the baseline evaluation of Asian women with PCOS [37], which should be linked to their long-term metabolic management. Our results show that a patient with a family history of diabetes rated her menstruation more problematic.

### Modifications of original PCOSQ

Another important contribution of this study is the enhancement of the face validity of the PCOSQ in its application in Chinese-speaking countries by incorporating five additional items into the original scale, presented as “Q14_2: frightened of getting diabetes,” “Q29: growth of visible acne,” “Q27: excess hair loss,” “Q30: feel acne is a problem,” and “Q28: excess hair loss is a problem”. The factor loadings of these new items were expected; Q27, Q28, Q29, and Q30 were loaded on the acne and hair loss domain and Q14_2 was loaded on the weight domain.

PCOS increases the risks of metabolic syndrome, insulin resistance, and diabetes, especially in Chinese patients [38], with elevated chronic inflammation markers such as interleckin-6 [39]. Asian women with PCOS at reproduction age are at risk of developing impaired glucose tolerance and type 2 diabetes [30]. Also, PCOS in Asian women is a significant risk factor of gestational diabetes [40]. In this regards, PCOS patients’ psychological disturbances due to the fear of developing diabetes may need to be concerned. It may be important to incorporate this issue into the study of HRQoL of Asian women affected by PCOS. However, the original PCOSQ only addresses PCOS patients’ concern about getting cancer, while their worries about the risk of getting diabetes is not assessed. In fact, our pilot participants rated the issue of developing diabetes more important than that of getting cancer and, overall, our study patients were a little more worried about having diabetes than cancer. Hence, it is important to understand PCOS patients’ emotional impairment due to their fear of getting diabetes. In this study, “afraid of getting diabetes” was loaded on the weight domain. PCOS is considered to be associated with weight, as many women with PCOS are overweight or obese, which makes them prone to getting diabetes [41]. As a result, PCOS patients, especially those with obesity, might be afraid of getting diabetes and present their underlying concerns or worry about their weight. In this regard, “afraid of getting diabetes” is considered acceptable to belong to the weight domain, although this item also represents an emotional disturbance.

Unlike the suggestions from previous studies that “tired easily” be included in the emotion domain, our results show that “tired easily” was loaded on the acne and hair loss domain. Because “tired easily” intuitively represents a type of emotional tiredness, it seems appropriate to be included in the emotion domain. However, previous studies showed that the factor loadings of this item on the emotion domain were borderline acceptable. For example, the loadings were 0.47 in its original development study [23], 0.39 in the validation study by Guyatt et al. [6], and 0.345 in another validation study by Jones et al. [21]. We further proposed possible reasons why “tired easily” was loaded on the acne and hair loss domain in this study. First, acne and hair loss (i.e., male-pattern hair loss) are important clinical presentations of PCOS, which are likely to provoke anxiety or make individuals feel self-conscious about their physical appearance [5]. In this regards, “tired easily” would represent patients’ being exhausted due to underlying acne or hair loss problems and is considered acceptable to be included in the acne and hair loss domain in the present study. Second, because most items in the emotion domain mention the term “PCOS”, while “tired easily” does not, there might be a wording effect in the emotion domain [42]. That is, the respondents may directly link their emotional disturbances to PCOS for the emotion domain, while they might not consider that “tired easily” is related to PCOS. As a result, “tired easily” performed better in the acne and hair loss domain than in the emotion domain.

Also, the lack of questions about acne may weaken the validity of the PCOSQ because acne is a common symptom of PCOS and leads to psychological disturbances in patients [21]. Hence, four acne items were incorporated into the mPCOSQ by Barnard et al. [5], which accounted for 10.38% of variance in their study. A difference between the mPCOSQ and the Chi-PCOSQ is the number of items related to acne problems. The mPCOSQ [5] has four items representing patients’ difficulties with acne problems: (i) To what extent was acne a problem for you in the last 2 weeks? (ii) In relation to your last menstruation, how much was acne a problem for you? (iii) How much time during the last 2 weeks did you feel unattractive because of acne? (iv) How much time during the last 2 weeks did you feel depressed as a result of acne? The first two items ask the extent of acne problems with different recall periods (e.g., in the last 2 weeks vs. the last menstruation) and the last two items focus on patients’ emotional disturbances (e.g., unattractive, depression) due to acne problem. Although the Chi-PCOSQ only contains two items associated with acne, it is believed that these two items are sufficient to understand acne-associated disturbances. This low number of items lowers the burden of participants. In addition, the acne-related items use “in the past 2 weeks” as the recall period, the same as that used for all remaining items in the scale.

Moreover, hair loss (androgenic alopecia, in a classic male pattern) as characterized by hyperandrogenism is another issue to be considered. Female-pattern baldness or hair loss is often associated with the development of PCOS and can have a significant psychological impact on a person, such as low self-esteem, depression, introversion, and feelings of unattractiveness. However, little research has assessed how such clinical presentation influences the HRQoL of women with PCOS. In fact, some of our pilot participants reported their concerns regarding hair loss and on average our study respondents felt moderate impairment due to this problem. This indicates the importance of incorporating the assessment of hair-loss-related items into the scale.

Furthermore, it has been recognized that racial and ethnic backgrounds may influence the impact of PCOS on HRQoL [25–28]. It is clear that symptoms commonly associated with PCOS, including acne, hirsutism, irregular menses, amenorrhea, obesity, and subfertility, are major sources of psychological and behavioral disorder, and that these clinical presentations vary in ethnic groups, which may result in different impact on HRQoL. One study showed that South Asian women with PCOS seemed not to have identifiably poorer HRQoL than did Caucasians with PCOS; the burden of hirsutism on HRQoL in South Asians was especially to a lesser extent than that in Caucasians, with weight and infertility as the worst domains on the condition-specific questionnaire for both ethnic groups [26]. Although women affected by PCOS commonly report excess hair growth (hirsutism), PCOS patients in China [35, 36] had low rates of hirsutism. Consistent with previous findings [35, 36], our Chinese respondents rated their concerns about the excess growth of body hair to a lesser extent, especially the aspect of “growth of visible hair on chin”.

Although the scoring approach in the Chi-PCOSQ is consistent with that in previous studies [6, 43] using the original PCOSQ, the difference in total number of items in the Emotional and Weight domains between the Chi-PCOSQ and the original PCOSQ may result in different domain and total scores between the two scales. Despite this difference, we found that the domain scores of Emotional and Weight domains in the present study were within the range of those in previous studies using original PCOSQ (Fig 1). An added Acne domain score seems to be higher in an Iranian study [24] which used the mPCOSQ [5], than that domain score of the Chi-PCOSQ in the present study (Fig 1). However, this difference may be attributable to ethnicity instead of the scoring method.

### Potential limitations

Several limitations of this study need to be addressed. First, the sample size was limited and all participants were from one medical center. Most of the participants had a high educational level and might have had good health literacy in terms of their self-care and/or health-seeking behaviors. It is crucial for future researchers to validate the Chi-PCOSQ using a large sample from different regions with various education and socioeconomic statuses. Although our sample size was based on a > 2:1 ratio of subjects to items [21, 34], others suggested that the ratio should be ≥ 5:1 [44]. Future studies containing such a ratio of respondents to items are recommended. Second, the translators for back translation were not native English speakers, which may affect the language fluency of the back-translated English versions of the PCOSQ. However, the back translation may be somewhat acceptable for the following reasons: (1) the concept of the back-translated English versions was consistent with the original scale; (2) the back translators had studied or worked in the United States for over 5 years, and (3) they had received high scores on the Test of English as a Foreign Language. Third, because our work was only a preliminary study, we did not have any priori anticipation for the magnitude of the correlations among measures. However, priori predictions are important for examining construct validity [6]. Therefore, we suggest that future studies need to have such priori anticipations before testing construct validity of the Chi-PCOSQ. Fourth, some of the results relating to factor analysis have produced item-domain linkages that some might find counter-intuitive (i.e., Q4: tiring easily loaded on the Acne&Hair loss domain in the present study). Future research is warranted to examine and verify our study results. Lastly, the responsiveness of the Chi-PCOSQ was not assessed in this study due to the limited study time period. Future research, along with sufficient follow-up time, should evaluate the ability of the Chi-PCOSQ to describe the health status of patients over time and its sensitivity to detect changes in HRQoL because of treatment or intervention, and determine the minimal clinical importance (MID) score to justify whether these changes are clinically relevant.

## Conclusion

The PCOSQ was translated into Chinese according to standard procedures. The Chi-PCOSQ demonstrated good measurement properties. In particular, three additional concepts, “acne,” “hair loss”, and “feel frightened of getting diabetes,” were incorporated and all showed good reliability and validity. Hence, the Chi-PCOSQ may better reflect the impact of PCOS on the HRQoL of patients. The HRQoL issues for women with PCOS have been little understood in East Asia and few comparison studies have explored the difference in the HRQoL of PCOS patients between Western and Eastern countries. This is in part due to the lack of a convincing tool specific to PCOS in Eastern culture. We recommend that the Chi-PCOSQ be used to improve our understanding of the health status of Chinese-speaking PCOS patients. The Chi-PCOSQ can potentially be used for Chinese-speaking women affected by PCOS; however, we suggest that additional studies be conducted to examine its psychometric properties for sub-cultures of Chinese-speaking populations (e.g., Hong Kong and different provinces of mainland China). Thus, the differences in PCOS attitudes among Chinese in different parts of the world can be examined and differences in HRQoL for PCOS patients across cultures can be identified. The longitudinal validity of the Chi-PCOSQ should be confirmed further in order to ensure its ability to address the symptoms and treatments for PCOS on HRQoL.

## Supporting Information

S1 Appendix Chinese Version of Polycystic Ovary Syndrome Health-Related Quality of Life Questionnaire (Chi-PCOSQ). (DOCX)
