# Supplementary material for: Development of Chinese Version of Polycystic Ovary Syndrome Health-Related Quality of Life Questionnaire (Chi-PCOSQ)
Source: PLoS One. 2015 Oct 9;10(10):e0137772. doi: 10.1371/journal.pone.0137772 (PMC4599828; doi:10.1371/journal.pone.0137772)
Supplement: S1 Appendix — (DOCX) [file pone.0137772.s001.docx]

**SUPPORTING INFORMATION**

**Appendix A: Chinese Version of Polycystic Ovary Syndrome Health-Related Quality of Life Questionnaire (Chi-PCOSQ)**

過去的兩個星期內，您覺得自己...

|  | 無時無刻覺得 | 總是覺得 | 常常覺得 | 有時候 覺得 | 偶而覺得 | 幾乎沒有 | 完全沒有 |
| --- | --- | --- | --- | --- | --- | --- | --- |
| 22. 因為過重而覺得自己不性感 | □ | □ | □ | □ | □ | □ | □ |
| 23. 覺得多囊性卵巢症候群的症狀無法控制 | □ | □ | □ | □ | □ | □ | □ |
| 24. 覺得維持理想的體重有困難 | □ | □ | □ | □ | □ | □ | □ |
| 25. 對於不孕感到難過 | □ | □ | □ | □ | □ | □ | □ |

過去的兩個星期內，您覺得下列問題對於您的困擾程度...

|  | 非常嚴重的困擾 | 嚴重的困擾 | 中等程度的困擾 | 些許這樣的困擾 | 一點點的困擾 | 幾乎沒有困擾 | 完全沒困擾 |
| --- | --- | --- | --- | --- | --- | --- | --- |
| 26. 身體有明顯的體毛 | □ | □ | □ | □ | □ | □ | □ |

過去的兩個星期內，您覺得下列問題對於您的困擾程度...

|  | 非常嚴重的困擾 | 嚴重的困擾 | 中等程度的困擾 | 些許這樣的困擾 | 一點點的困擾 | 幾乎沒有困擾 | 完全沒困擾 |
| --- | --- | --- | --- | --- | --- | --- | --- |
| 15. 臉上有明顯的細毛 | □ | □ | □ | □ | □ | □ | □ |
| 16. 對於過多的體毛感到尷尬 | □ | □ | □ | □ | □ | □ | □ |

過去的兩個星期內，您覺得自己...

|  | 無時無刻覺得 | 總是覺得 | 常常覺得 | 有時候 覺得 | 偶而覺得 | 幾乎沒有 | 完全沒有 |
| --- | --- | --- | --- | --- | --- | --- | --- |
| 17. 擔心自己患有多囊性卵巢症候群 | □ | □ | □ | □ | □ | □ | □ |
| 18.因患有多囊性卵巢症候群而變敏感 (例如:覺得容易成為被注視或討論的對象) | □ | □ | □ | □ | □ | □ | □ |

最後一次月經週期時，您覺得下列問題對於您的困擾程度...

|  | 非常嚴重的困擾 | 嚴重的困擾 | 中等程度的困擾 | 些許這樣的困擾 | 一點點的困擾 | 幾乎沒有困擾 | 完全沒困擾 |
| --- | --- | --- | --- | --- | --- | --- | --- |
| 19. 腹脹 | □ | □ | □ | □ | □ | □ | □ |
| 20. 月經延遲 | □ | □ | □ | □ | □ | □ | □ |
| 21. 經痛 | □ | □ | □ | □ | □ | □ | □ |

最後一次月經週期時，您覺得下列問題對於您的困擾程度...

|  | 非常嚴重的困擾 | 嚴重的困擾 | 中等程度的困擾 | 些許這樣的困擾 | 一點點的困擾 | 幾乎沒有困擾 | 完全沒困擾 |
| --- | --- | --- | --- | --- | --- | --- | --- |
| 7. 頭痛 | □ | □ | □ | □ | □ | □ | □ |
| 8. 月經週期不規則 | □ | □ | □ | □ | □ | □ | □ |

過去的兩個星期內，您覺得下列問題對於您的困擾程度...

|  | 非常嚴重的困擾 | 嚴重的困擾 | 中等程度的困擾 | 些許這樣的困擾 | 一點點的困擾 | 幾乎沒有困擾 | 完全沒困擾 |
| --- | --- | --- | --- | --- | --- | --- | --- |
| 9. 上唇鬍子明顯增加 | □ | □ | □ | □ | □ | □ | □ |

過去的兩個星期內，您覺得自己...

|  | 無時無刻覺得 | 總是覺得 | 常常覺得 | 有時候 覺得 | 偶而覺得 | 幾乎沒有 | 完全沒有 |
| --- | --- | --- | --- | --- | --- | --- | --- |
| 10. 對於自己的體重控制有困難 | □ | □ | □ | □ | □ | □ | □ |
| 11. 因患有多囊性卵巢症候群而自卑 | □ | □ | □ | □ | □ | □ | □ |
| 12. 在減重過程中感到挫折 | □ | □ | □ | □ | □ | □ | □ |
| 13. 害怕可能無法生育 | □ | □ | □ | □ | □ | □ | □ |
| 14. 害怕可能得到癌症 | □ | □ | □ | □ | □ | □ | □ |

過去的兩個星期內，您覺得下列問題對於您的困擾程度...

|  | 非常嚴重的困擾 | 嚴重的困擾 | 中等程度的困擾 | 些許這樣的困擾 | 一點點的困擾 | 幾乎沒有困擾 | 完全沒困擾 |
| --- | --- | --- | --- | --- | --- | --- | --- |
| 1.下巴鬍鬚明顯增加? | □ | □ | □ | □ | □ | □ | □ |

過去的兩個星期內，您覺得自己...

|  | 無時無刻覺得 | 總是覺得 | 常常覺得 | 有時候 覺得 | 偶而覺得 | 幾乎沒有 | 完全沒有 |
| --- | --- | --- | --- | --- | --- | --- | --- |
| 2. 因為患有多囊性卵巢症候群而感到憂鬱 | □ | □ | □ | □ | □ | □ | □ |
| 3. 擔心體重過重 | □ | □ | □ | □ | □ | □ | □ |
| 4. 容易疲倦 | □ | □ | □ | □ | □ | □ | □ |
| 5. 擔心不孕 | □ | □ | □ | □ | □ | □ | □ |
| 6. 因為患有多囊性卵巢症候群而感到情緒不穩定 | □ | □ | □ | □ | □ | □ | □ |
